# Identification of CD137-Expressing B Cells in Multiple Sclerosis Which Secrete IL-6 Upon Engagement by CD137 Ligand

**DOI:** 10.3389/fimmu.2020.571964

**Published:** 2020-11-06

**Authors:** Hiu Yi Wong, Ankshita Prasad, Shu Uin Gan, John Jia En Chua, Herbert Schwarz

**Affiliations:** ^1^ Department of Physiology, National University of Singapore, Singapore, Singapore; ^2^ Immunology Programme, Yong Loo Lin School of Medicine, National University of Singapore, Singapore, Singapore; ^3^ Institute for Health Innovation and Technology, National University of Singapore, Singapore, Singapore; ^4^ Department of Surgery, National University of Singapore, Singapore, Singapore; ^5^ LSI Neurobiology Programme, National University of Singapore, Singapore, Singapore

**Keywords:** multiple sclerosis, neuroinflammation, CD137, B cell, IL-6

## Abstract

The potent costimulatory effect of CD137 has been implicated in several murine autoimmune disease models. CD137 costimulates and polarizes antigen-specific T cells toward a potent Th1/Tc1 response, and is essential for the development of experimental autoimmune encephalomyelitis (EAE), a murine model of Multiple Sclerosis (MS). This study aimed to investigate a role of CD137 in MS. Immunohistochemical and immunofluorescence staining of MS brain tissues was used to identify expression of CD137. CD137^+^ cells were identified in MS brain samples, with active lesions having the highest frequency of CD137^+^ cells. CD137 expression was found on several leukocyte subsets, including T cells, B cells and endothelial cells. In particular, CD137^+^ B cells were found in meningeal infiltrates. *In vitro* experiments showed that CD137 engagement on activated B cells increased early TNF and persistent IL-6 secretion with increased cell proliferation. These CD137^+^ B cells could interact with CD137L-expressing cells, secrete pro-inflammatory cytokines and accumulate in the meningeal infiltrate. This study demonstrates CD137 expression by activated B cells, enhancement of the inflammatory activity of B cells upon CD137 engagement, and provides evidence for a pathogenic role of CD137^+^ B cells in MS.

## Introduction

CD137 [TNFRSF9, 4-1BB, induced by lymphocyte activation (ILA)] is a member of the tumor necrosis factor (TNF) receptor family (1, 2). It is transiently expressed on T cells upon activation. CD137 binds to its ligand, CD137L, which is expressed on antigen presenting cells (APCs) such as dendritic cells, macrophages and B cells. CD137 signaling co-stimulates activated antigen-specific T cells, and enhances immune responses (3). At the same time, CD137L reverse signaling into CD137L-expressing APC and induces proliferation, differentiation and maturation, which enhances their antigen-presenting capacity (4).

The potent costimulatory effect of CD137 is implicated in autoimmunity (5). Besides activated T cells, CD137 is also expressed by activated natural killer (NK) cells (6, 7), neutrophils (8) and vascular endothelial cells (9). CD137/CD137L signaling enhances inflammation and increases recruitment of monocytes to sites of inflammation. Soluble CD137 (sCD137), which is generated by differential splicing, is found at elevated levels in sera of patients with autoimmune diseases, including rheumatoid arthritis (RA) and Multiple Sclerosis (MS) (10, 11).

In particular, the role of CD137 in MS is worth investigating. MS is a neuroinflammatory disease affecting more than 2.3 million people worldwide (12). By yet unknown causes, autoreactive T and B cells against myelin are activated. Disruption of the blood brain barrier (BBB) and subsequent migration of peripheral inflammatory monocytes, T and B cells into the central nervous system (CNS) results in inflammation and demyelination (13). Lesions in MS are characterized by demyelination with axonal loss, macrophage and microglia activation and gliosis (14). As CD137 signaling strongly costimulates antigen-specific T cells and polarizes them toward a potent Th1/Tc1 response (15), it has the potential to contribute to the inflammation in MS. Furthermore, the enhanced antigen presenting capacity induced by CD137L signaling in macrophages likely applies to microglia as well, which are the tissue-resident macrophages in the CNS (4).

Previous studies identified CD137 and CD137L to be expressed in the human CNS, and their expression increased during inflammation caused by mycobacterial infection (16). Furthermore, the presence of CD137L-activated murine microglia induced apoptosis of oligodendrocytes, and led to a significant decrease in oligodendrocyte population when compared to that of non-activated microglia (17). These *in vitro* data were further supported by the experimental autoimmune encephalitis (EAE) model. CD137L^-/-^ mice were largely protected from EAE while wild type (WT) mice succumbed to the disease. There was a reduced infiltration of myeloid cells and CD137^+^ T cells into the CNS, and less spinal cord demyelination in CD137L^-/-^ than in WT mice (18). Based on the *in vitro* and *in vivo* evidence, we investigated the potential role of CD137 in the pathogenesis of MS, using post-mortem brain sections from MS patients and *in vitro* experiments on human immune cells. We find that CD137 is expressed by several leukocyte subsets, with CD137^+^ B cells found in meningeal infiltrates. CD137 signaling into B cell lines and primary B cells induces secretion of TNF and IL-6 with increased cell proliferation. These identify potential mechanisms how CD137^+^ B cells may augment inflammation in autoimmune diseases.

## Materials and Methods

### Patient Samples

Formalin-fixed and paraffin-embedded (FFPE) sections were used. Human tonsil FFPE sections, which served as positive controls for staining of immune markers, were obtained from the Department of Pathology of the National University Hospital, Singapore with approval from the Institutional Review Board, Singapore (IRB number: B16-309). MS brain tissue FFPE samples and associated clinical and neuropathological data were supplied by the Multiple Sclerosis Society Tissue Bank, funded by the Multiple Sclerosis Society of Great Britain, registered charity 207495 (authorized by the Wales MREC to release tissue samples for research). Slides with samples from post-mortem brains were from different patients (n = 36) as well as healthy individuals (n = 8). For some patients, sections with more than one type of lesion were obtained. Sections included various types of MS lesions, namely normal appearing white matter (NAWM) lesions (n = 12), active lesion (AL) (n = 9), chronic active lesions (CAL) (n = 12), chronic lesions (CL) (n = 10), remyelinating lesions (RL) (n = 11) and healthy controls (HC) (n = 8), as classified according to Li et al (19). Three samples with significant meningeal infiltrates were also studied. Characteristics of MS patients and healthy controls are depicted in **Supplementary Table 1**, while **Supplementary Figure 1** shows the staining of immune markers in human tonsils. Information regarding treatment received by many patients was limited.

### Immunohistochemical and Immunofluorescence Staining

To determine the extent of demyelination, Luxol fast blue & Cresyl staining was performed according to manufacturer’s protocol (Sigma-Aldrich, MO, USA). Images were obtained using Leica DM2000 (Leica Microsystems, Wetzlar, Germany). Opal multiplex immunofluorescent system (Opal staining) was adopted for multi-colour staining (Perkin Elmer, Waltham, USA). Staining was performed according to manufacturer’s protocol and optimal concentrations of primary antibodies were predetermined using human brain and tonsil tissues. Primary antibodies used in the Opal staining included mouse anti-human CD137 (clone: BBK2, Thermo Fisher Scientific, Waltham, USA), rabbit anti-human CD3 (Dako, Santa Clara, USA), rabbit anti-human CD19 (clone: 2E2B6B10; Abcam, Cambridge, UK), rabbit anti-human CD45 (Abcam), and rabbit anti-human ionized calcium-binding adapter molecule-1 (Iba-1) (FUJIFILM Wako Pure Chemical Corporation, Osaka, Japan). Secondary antibodies consisted of horseradish peroxidase (HRP) labeled antibodies against rabbit and mouse (GBI Labs, Bothell, USA). Images were taken and analyzed using the Vectra microscope system (Perkin Elmer) and inForm^®^ Cell Analysis^™^ (Perkin Elmer) at the Cancer Science Institute of Singapore and the Genome Institute of Singapore, A*STAR. For each section, 40 to 150 random images were taken depending on the size of section. Number of cells per image were counted both manually and by inForm® software. The mean number of respective cells was calculated per mm^2^ of section for each patient/control sample. Multiplex and respective single colour staining of CD137, CD3, and CD19 in human tonsil served as positive controls (**Supplementary Figure 1**). **Supplementary Figure 2** shows CD137 and CD3 double immunohistochemical staining on tonsil with respective isotype controls.

### Transduction of Cells

DG-75 and BJAB are human Burkitt lymphoma cell lines with no constitutive CD137 expression, and were purchased from the German Collection of Microorganisms and Cell Cultures (DSMZ; Braunschweig, Germany). Full length cDNA of CD137 was cloned into pLenti6 (full name of the vector as in catalog) vector (Invitrogen, Carlsbad, CA), and lentiviral particles were produced with Lenti-vpak packaging kit (Origene, Maryland, USA). DG-75 and BJAB cells were transduced and selected with medium containing 7.5 μg/ml and 5 μg/ml of Blasticidin, respectively. Parental (DG-75 and BJAB) and transduced (DG75-CD137 and BJAB-CD137) cells were cultured in RPMI 1640 (Biowest, Utah, USA) supplemented with 10% fetal bovine serum (FBS).

### Cell Stimulation and Co-Culture

All blood samples were obtained from healthy donors at Health Sciences Authority Singapore with approval from the Institutional Review Board, Singapore (IRB number: 13-079E) in accordance to the guidelines of the Health Sciences Authority of Singapore. Peripheral blood mononuclear cells (PBMCs) were prepared by Ficoll-Pague (GE Healthcare, Chicago, USA) density gradient centrifugation. B cells were isolated from PBMCs by positive selection using EasySep™ Human CD19 Positive Selection Kit (STEMCELL Technologies, BC, Canada). The purity of isolated B cell population was over 95%. The remaining PBMCs from the same donor were stimulated with 100 ng/ml of anti-human CD3 antibody (clone OKT3; Biolegend, San Diego, USA) at a density of 5 x 10^6^ cells/ml for 72 h to generate activated T cells. All cells were maintained in RPMI 1640 (Biowest) supplemented with 10% FBS, 50 μg/ml streptomycin and 50 IU/ml penicillin.

To identify a method to induce CD137 expression on B cells, isolated B cells were stimulated with either 25 ng/ml PMA + 1 μg/ml of ionomycin, 5 μg/ml of PHA, 5 μg/ml of LPS, 1000 IU/ml of IL-2 + 5 μg/ml of R848 ± 1 μg/ml of anti-human CD40 antibody (eBioscience, CA, USA) or 1 μg/ml of anti-human CD40 antibody + 10 μg/ml of AffiniPure F(ab’)_2_ Fragment Goat Anti-Human IgA + IgG + IgM (H+L) (Jackson ImmunoResearch Laboratories, INC, PA, USA) at a density of 10^6^ cells/ml (20, 21).

B cell lines and stimulated primary B cells were cultured in tissue culture plates coated with 5 μg/ml of recombinant human CD137L (R&D Systems, Minneapolis, MN) in phosphate-buffered saline (PBS) or 5 μg/ml of bovine serum albumin (BSA) in PBS for 24 h. To determine cell proliferation, B cells were first labeled with CellTrace™ Cell Proliferation Kit (Thermo Fisher Scientific), followed by stimulation as described above, and cultured in tissue culture plates coated with 5 μg/ml recombinant human CD137L or BSA in PBS for 7 days. Cells were then harvested for flow cytometry and supernatants were stored at -80°C.

### Flow Cytometry

Cells were stained with PE-conjugated anti-human CD137 (BD Pharmingen, San Jose, USA), PE-conjugated anti-human CD40 (eBioscience), PE-conjugated anti-human B cell activating factor receptor (BAFFR) (eBioscience) and APC-conjugated anti-human CD86 (Biolegend) antibodies. The isotype controls were mouse-IgG1k with respect to fluorophore-conjugated antibodies. To determine cell viability and proliferation, cells were stained with LIVE/DEAD™ Fixable Near-IR Dead Cell Stain Kit (Life Technologies, CA, USA). Viable B cells were gated to determine CFSE dilution. All results were obtained using Fortessa Analyser (BD Biosciences, New Jersey, USA) and analyzed using FlowJo software (FlowJo, LLC, Ashland, USA).

### ELISA

Levels of TNF, IL-6, IL-8, IL-10, and GM-CSF in supernatants were measured using the respective ProcartaPlex Immunoassays (Thermo Fisher Scientific) according to manufacturer’s protocol and all measurements were performed in duplicates. Readings were analyzed using MAGPIX^TM^ System with xPonent^TM^ 4.2 software (Thermo Fisher Scientific).

### Reverse Transcriptase Polymerase Chain Reaction

Stimulated B cells and T cells were harvested and total RNA were extracted using RNeasy Mini Kit (Qiagen, Hilden, Germany). cDNA was synthesized using random primers (Thermo Fisher Scientific) followed by reverse transcriptase polymerase chain reaction (RT-PCR) using forward and reverse primers encoding full length CD137 (Thermo Fisher Scientific). Purified cDNA products were run on 1.5% agarose gel electrophoresis and the gel was then visualized using Azure c600 (Azure Biosystems, Dublin, USA).

### Organoid Culture and Immunostaining

The cerebral organoids were derived using the STEMdiff™ Cerebral Organoid Kit (STEMCELL Technologies) according to the manufacturer’s protocol and to published literature (22). Briefly, human embryonic stem cells were cultured in mTeSR™ (STEMCELL Technologies) and disassociated using Gibco™ Accutase (Thermo Fisher Scientific). To generate embryonic bodies (EBs), 9,000 cells per well was seeded in a low attachment 96 well- plate containing EB formation media. On day 5, the EBs were cultured in Neural Induction media for two days following which they were embedded in 15 µl of ESC qualified Matrigel (Corning, NY, USA), and cultured in Expansion medium to generate neuroepithelial rosettes. From day 10 onwards, the organoids were grown on an orbital shaker placed in the cell culture incubator.

DG-75 and DG75-CD137 cells were first labeled with CellTrace™ CFSE Cell Proliferation Kit (Thermo Fisher Scientific). Organoids (D42) were then co-cultured with labeled DG-75 or DG75-CD137 cells (300,000 cells in 2 ml of media). Organoids were harvested after 48 h of co-culture. Organoid without DG-75 co-culture served as negative control.

Harvested organoids were fixed with 4% PFA in PBS (2.7 mM KCl, 1.5 mM KH_2_PO_4_, 137 mM NaCl, 8 mM Na_2_HPO_4_, ddH_2_O, pH 7.3), (3 h at RT) and permeabilized using 1% Triton-X100 (72 h at RT). They were subsequently blocked using blocking buffer containing 2% BSA and 1% Triton-X100 in PBS (48 h at 4°C). Organoids were then incubated with primary antibody diluted in blocking buffer (Tau, Guinea Pig, 1:400) for 72 h. The organoids were subsequently washed 3X in washing buffer containing 2% BSA, 3% NaCl and 0.2% Triton-X100 in PBS and incubated with secondary antibodies (Cy5, 1:200, Synaptic Systems, Goettingen, Germany) for 72 h. Organoids were washed 3X with PBS and incubated with RapiClear 1.52 (Sunjin Lab, Hsinchu City, Taiwan) for optical clearing (2 h at RT). The organoids were mounted on a glass slide with 0.5 mm chambers filled with RapiClear. Imaging was performed on a Zeiss confocal microscope and images were analyzed using the IMARIS software (Bitplane, Belfast, United Kingdom).

### Statistical Analysis

Statistical significance was determined by Pearson correlation, Student *t*-test and one-way ANOVA with Bonferroni comparison using GraphPad Prism software (San Diego, CA, USA). A *p* value of <0.05 was considered as significant.

## Results

### CD137^+^ Cells Identified in MS Lesions, With Highest Frequency in Active Lesions

**Figure 1:**
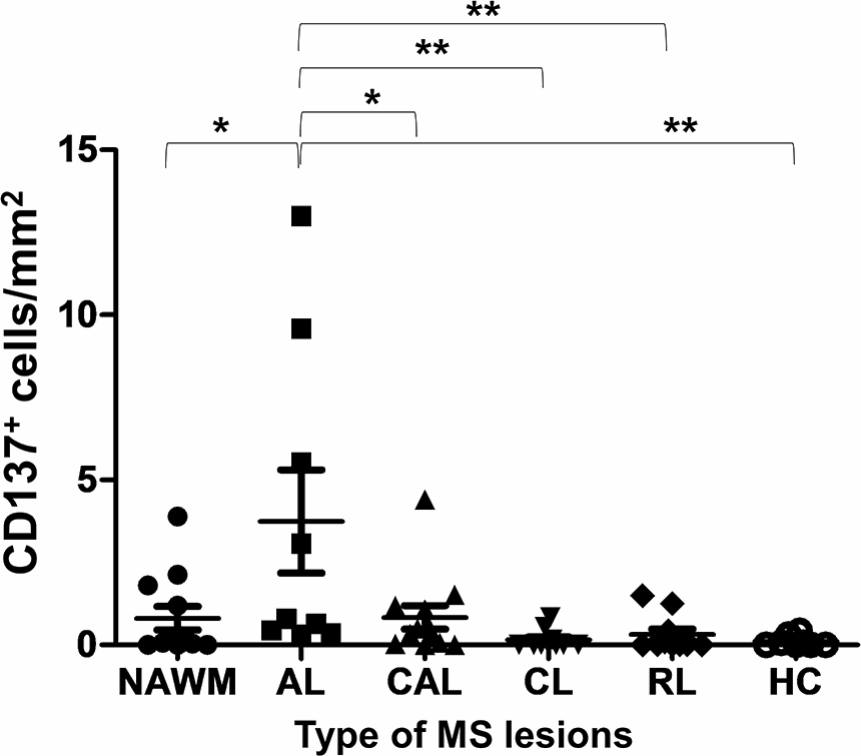
Number of CD137^+^ cells in different types of MS lesions and healthy controls. Brain sections of different type of MS lesions and healthy controls were stained for CD137 expression using multiplex immunofluorescence staining. One section per donor was stained for CD137 expression. For each section, 40 to 150 random images were taken depending on the size of section. Number of cells per image were counted both manually and by inForm® software. The mean number of CD137^+^ cells was calculated per mm^2^ of section for each patient/control sample. Results are pooled from all stained sections and illustrated as means ± standard errors. NAWM, normal appearing white matter lesions (n = 12); AL, active lesions (n = 9); CAL, chronic active lesions (n = 12); CL, chronic lesions (n = 10); RL, remyelinating lesions (n = 11); HC, healthy controls (n = 8). *p < 0.05 and **p < 0.001 using one-way ANOVA with Bonferroni comparison.

A total of 54 MS tissue sections and eight normal control tissues were analyzed. CD137^+^ cells were found in 38 samples, and in all types of MS lesions. CD137 expression was also found in healthy controls, though the number of CD137^+^ cells was scarce (**Supplementary Figure 3**). There was no correlation between the number of CD137^+^ cells and age at death, duration of disease and sex of patients (p = 0.15, 0.58, and 0.46, respectively). Significantly more CD137^+^ cells were found in active MS lesions than in other lesion types (**Figure 1**). Active lesions are classified as having significant inflammatory infiltrates with demyelination (14). This observation is expected since CD137 expression is activation-dependent. The higher number of CD137^+^ cells in active lesions indicates a potential role of CD137 signaling in the inflammatory response during the acute inflammatory stage of disease.

**Figure 2:**
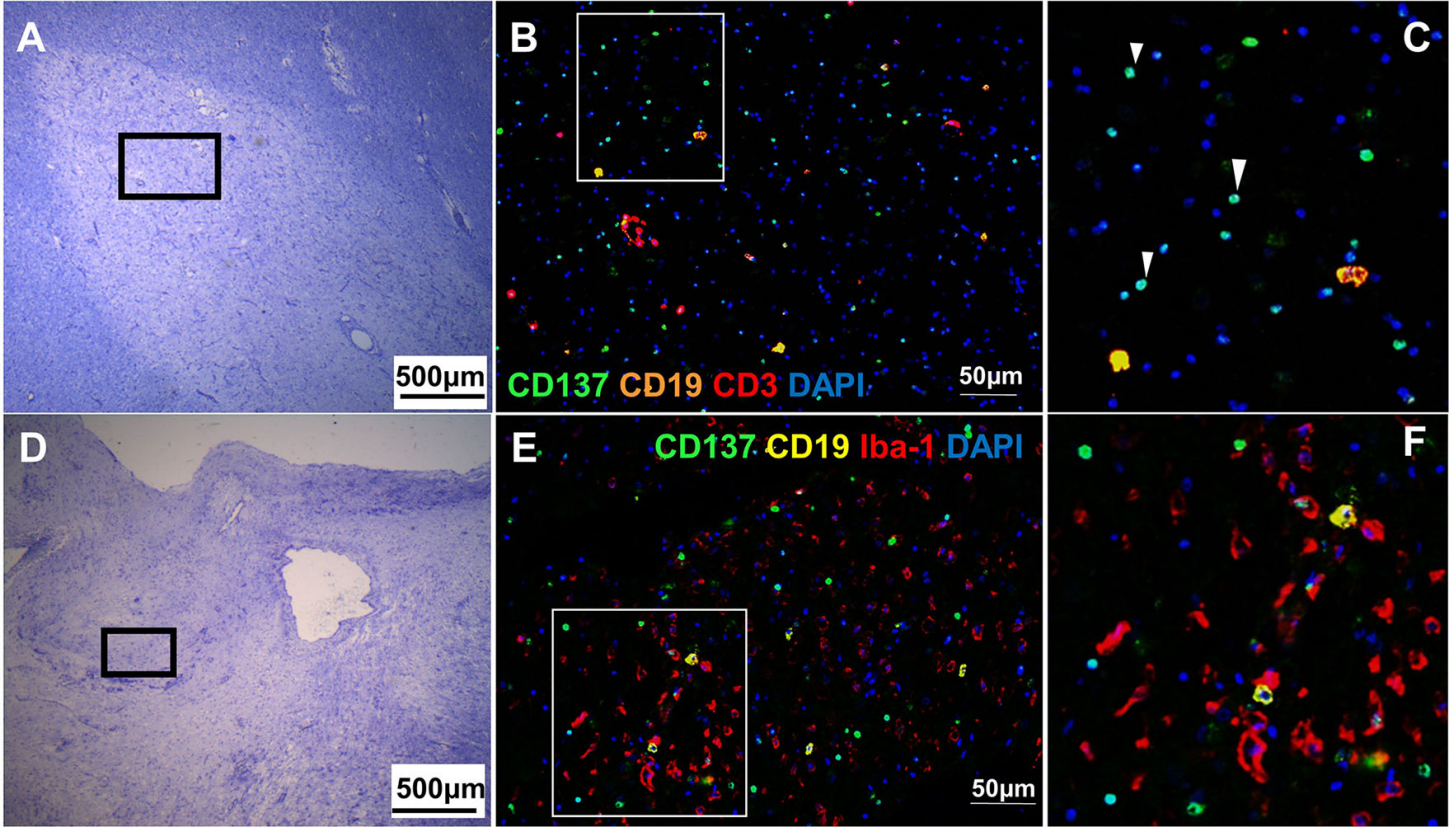
CD137^+^ cells adjacent to inflammatory cells in active MS lesions. Representative images from two donors (MS 058 and MS 180) are shown. **(A)** Luxol fast blue and Cresyl staining of a section from orbitofrontal cortex with an active lesion (MS 058). Discolouration indicates demyelination. **(B, C)** Opal staining of boxed region in A. Many CD137^+^ cells (white arrowheads) were found together with T cells and B cells. **(D)** Luxol fast blue and Cresyl staining of a section from a temporal lobe with an active lesion (MS 180). **(E, F)** Opal staining of boxed region in **(D)**. Diffuse macrophage and microglial infiltration (red) was seen with many CD137^+^ cells (green). For each donor, images were obtained from one section in one experiment.

**Figure 3:**
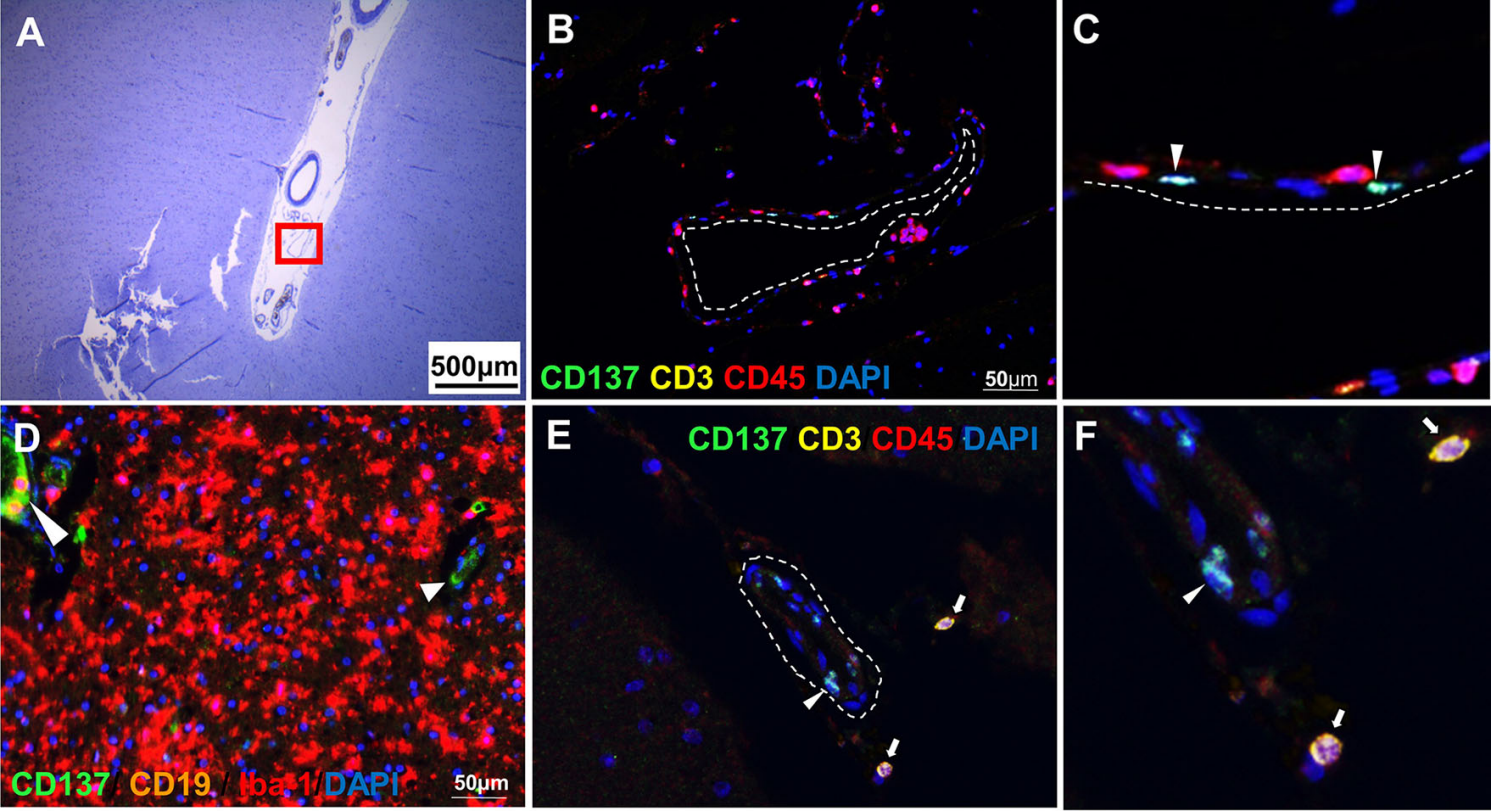
CD137^+^ cells in blood vessels. Representative images from two donors (MS 325 and MS 492) are shown. **(A)** Luxol fast blue and Cresyl staining of a section from the temporal lobe with an active lesion (MS 325). **(B, C)** Opal staining of the boxed region in A (red square). CD137^+^ cells were found along the endothelial lining (white dotted line) of blood vessels (white arrowheads). **(D)** Another area within the parenchyma of active lesion (MS 325). CD137 expression was shown on the endothelial lining of capillaries (white arrowheads). **(E, F)** CD137 expression (white arrowhead) on a blood vessel wall in a normal appearing white matter lesion (MS 492) with infiltrating CD3^+^ CD45^+^ T cells (white arrows). For each donor, images were obtained from one section in one experiment.

CD137^+^ cells were commonly found in the perivascular regions, together with neighboring T and B cells (**Figure 2**). Abundant CD137^+^ cells were found adjacent to B and T cells in an active lesion from orbitofrontal cortex (**Figures 2A–C**). Diffuse macrophage and microglia infiltration (red in colour) was observed in the demyelinating area with many CD137^+^ cells (**Figures 2D–F**). Besides in the brain parenchyma, CD137^+^ cells were also found on blood vessel walls in different types of MS lesions, but not in healthy controls. Further, CD137^+^ cells could be seen along the lining of the endothelium (**Figure 3**). These cells were found with CD45^+^ cells in the endothelium (**Figures 3B, C**) next to T cells (**Figures 3E, F**). They can be found in all types of MS lesions, particularly in active lesions. Expression of CD137 has been documented on inflamed vascular endothelium before and has been associated with extravasation of leukocytes (9, 23).

CD137^+^ CD3^+^ cells were present in seven samples, although at low numbers (5–10 cells per section). CD137^+^ CD45^+^ cells were found in seven samples. Since no Iba-1^+^ macrophages and microglial cells were found to express CD137, these CD137^+^ CD45^+^ CD3^-^ leukocytes may be NK cells, B cells or neutrophils (**Supplementary Figure 4**).

### CD137-Expressing B Cells Identified in MS Active Lesions and Meningeal Infiltrates

CD137^+^ CD19^+^ B cells were found in two MS patients with active lesions and in one patient with diffuse chronic lesions and meningeal infiltrates. CD137 expression on T cells, NK cells and vascular endothelial cells has been well documented but not on human APCs (3, 7, 9). Two previous studies have identified CD137 expression on activated B cells of healthy donors, and on B cells of patients suffering from chronic lymphocytic leukemia (CLL) (20, 24). This is the first time CD137^+^ CD19^+^ B cells to be identified in an autoimmune disease.

**Figure 4:**
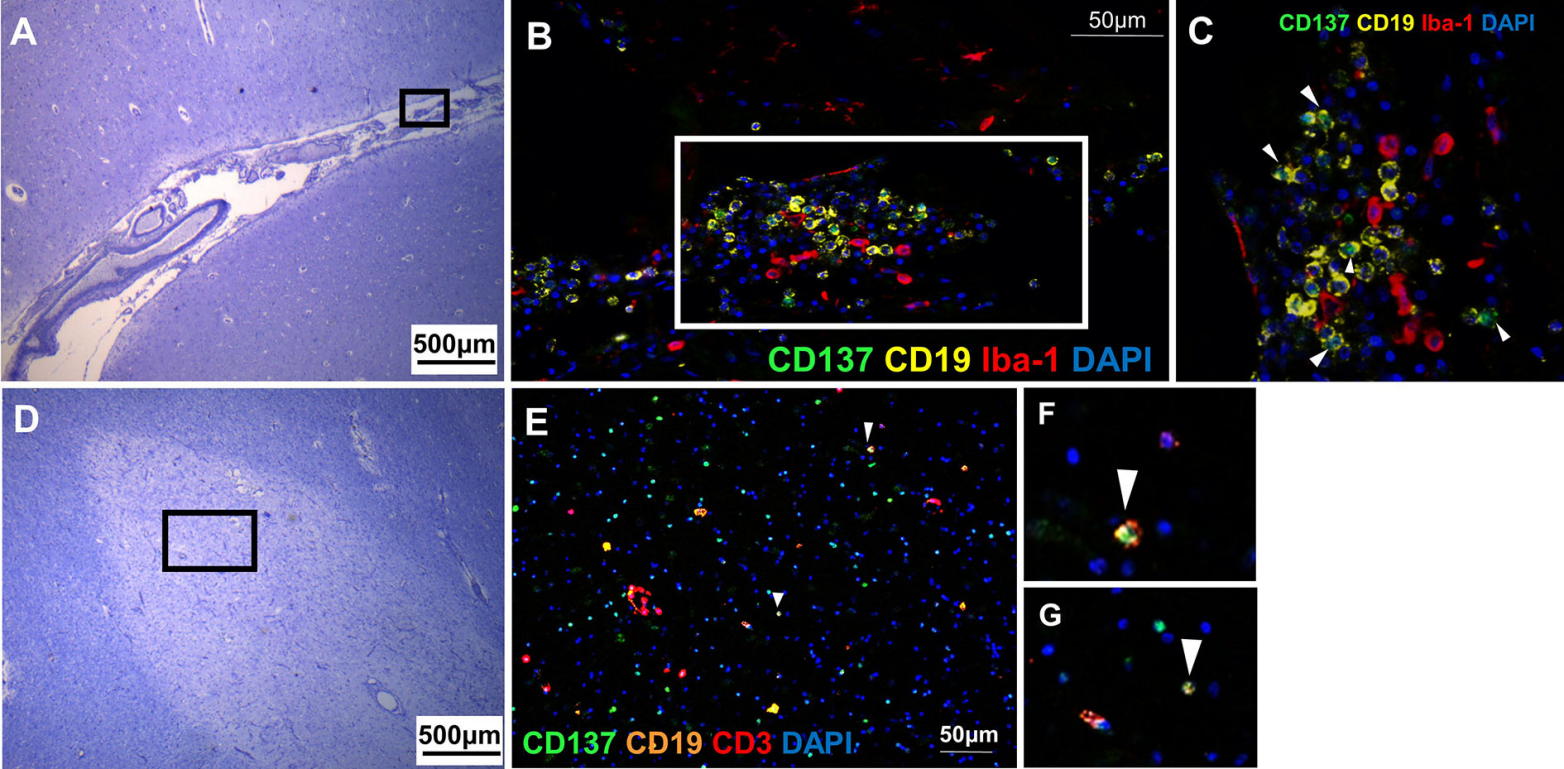
CD137^+^ B cells in MS active lesions and meningeal infiltrates. Representative images from two donors (MS 058 and MS 180) are shown. **(A)** Luxol fast blue and Cresyl staining of a section with moderate leptomeningeal inflammation (MS 180). **(B)** Opal staining of the boxed region in A. CD137^+^ CD19^+^ B cells were found among the leptomeningeal infiltrates. **(C)** Magnified view of boxed region in B. CD137^+^ CD19^+^ B cells (white arrowheads) were found together with neighboring Iba-1^+^ macrophages. **(D)** Luxol fast blue and Cresyl staining of a section from orbitofrontal cortex with active lesion (MS 058). **(E–G)** Opal staining of boxed region in **(D)**. Cells co-expressing CD137 and CD19 indicative of B cells were found (white arrowheads). For each donor, images were obtained from one section in one experiment.

CD137^+^ B cells were present in an active lesion and the meningeal infiltrate of a MS lesion with moderate leptomeningeal inflammation. CD137^+^ cells clustered with Iba-1^+^ macrophages (**Figures 4A–C**). CD137^+^ B cells were also found in active inflammatory lesions (**Figures 4D–G**).

B cells are known to act as APC to co-stimulate activated T cells (25). It has been shown that CD137L reverse signaling into B cells leads to proliferation and differentiation of B cells (26). However, knowledge about how CD137 expression is induced on B cells, and how CD137 signaling into B cells participates in the inflammatory process is limited. Since we identified CD137^+^ B cells in MS lesions, we proceeded to functional experiments to delineate the role of CD137 signaling into B cells, and their potential importance in augmenting inflammation.

### CD137 Signaling in B Cell Lines Induced Cell Activation and Cytokine Secretion

**Figure 5:**
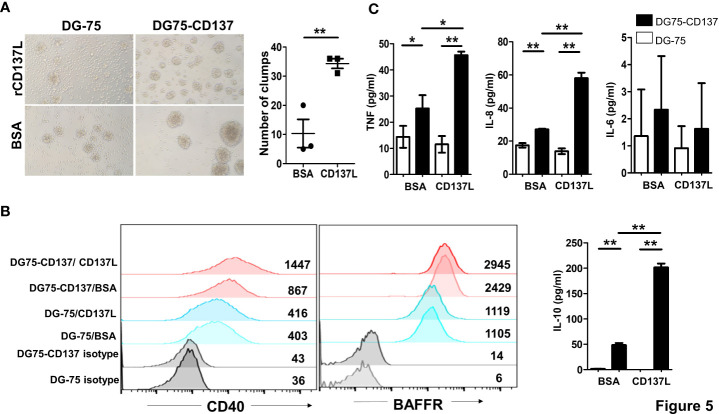
CD137 signaling into B cell lines induces cell activation and cytokine secretion. **(A)** Cells were cultured in plates coated with 5 μg/ml of either rCD137L protein or BSA for 24 h. BSA-coated plates served as negative controls. Number of clumps in rCD137L and BSA treated DG75-CD137 cells were counted. One clump is defined as more than five cells aggregated. Lines and bars are illustrated as means ± standard errors and results are pooled from three independent experiments. Each symbol represents one measurement. **(B)** Increased CD40 expression was found on DG75-CD137 cells upon rCD137L activation when compared to BSA-treated DG75-CD137 cells. Representative images and flow cytometry results of one experiment is shown. Numbers in the chart represent mean fluorescence index of the respective cell populations. To the right of the histograms, results of three independent experiments are quantified. Lines and error bars depict means ± standard errors. **(C)** Significantly higher levels of TNF, IL-8, and IL-10 were secreted upon rCD137L activation for DG75-CD137 cells compared to controls. Lines and bars are illustrated as means ± standard deviations. Results are pooled from three independent experiments and all measurements were in duplicates. *p < 0.05 and **p < 0.001 using two-tailed unpaired t test.

In order to understand the role of CD137 signaling into B cells, we generated two CD137^+^ B cell lines (DG75-CD137 and BJAB-CD137), using lentiviral transduction (**Supplementary Figure 5**). Upon activation by recombinant, plate-bound CD137L protein (rCD137L), DG75-CD137 cells formed small clumps and showed increased adherence to the plate compared to DG-75 control cells. Significantly more clumps were observed in DG75-CD137 cells treated with CD137L (**Figure 5A**). Expression of CD40 in rCD137L-activated DG75-CD137 cells was increased while BAFFR expression remained unchanged (**Figure 5B**). DG75-CD137 cells secreted enhanced levels of TNF, IL-8, and IL-10 which was further increased when the cells were activated with rCD137L (**Figure 5C**). Consistent results were obtained with BJAB-CD137 cells (**Supplementary Figure 6**). These results suggest that CD137 signaling into B cells induces or enhances cell activation, evidenced by increases in cell aggregation/adhesion, expression of activation markers and release of cytokines.

### CD137 Expression Induced Upon B Cell Receptor Activation and CD40 Signaling

**Figure 6:**
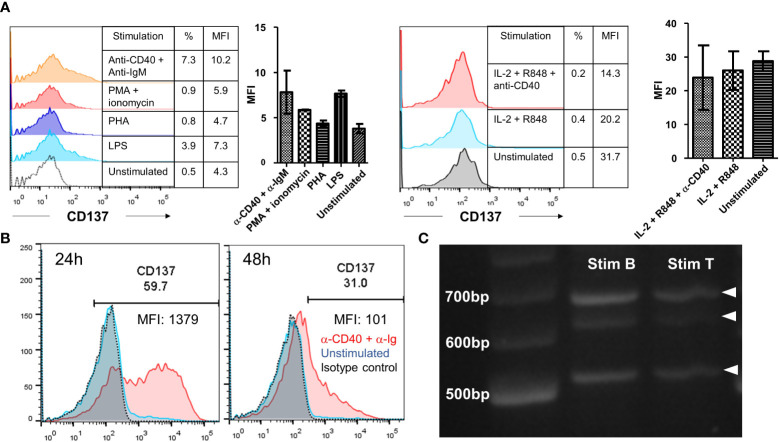
Induction of CD137 expression on primary B cells. **(A)** Isolated B cells from healthy donors were stimulated by either 25 ng/ml PMA + 1 μg/ml of ionomycin, 5 μg/ml of PHA, 5 μg/ml of LPS, 1 μg/ml of anti-human CD40 antibody + 10 μg/ml of anti-IgM antibody or 1000 IU/ml of IL-2 + 5 μg/ml of R848 ± 1 μg/ml of anti-CD40 antibody for 24 h. Cells were then harvested for flow cytometry measuring the expression of CD137. Lines and bars depict means ± standard errors and results are pooled from two different donors. **(B)** Isolated B cells from healthy donors were stimulated with 1 μg/ml of anti-human CD40 antibody + 10 μg/ml of AffiniPure F(ab’)_2_ Fragment Goat Anti-Human IgA + IgG + IgM (H+L) for up to 48 h. Cells were then harvested for flow cytometry at 24 h and 48 h. The same experiment was repeated for five different donors with consistent results. Representative results from one donor are shown. **(C)** RT-PCR products generated from RNA of stimulated B and T cells. 20 ng/lane of purified RT-PCR products from stimulated B and T cells were run using gel electrophoresis. Representative image from one donor is shown and experiment has been repeated with two donors with consistent results. %: percentage of CD137^+^ cells, MFI, mean fluorescence index.

General cellular activators, such as PHA and ionomycin could not induce CD137 expression on primary B cells. A small but non-significant induction of CD137 expression was observed upon activation by LPS or anti-IgM with anti-CD40 antibodies (**Figure 6A**). However, we observed induction of CD137 expression upon activation by antibodies against CD40 and human immunoglobulins with peak expression from 24 to 48 h after stimulation (**Figure 6B**).

RNA extracted from stimulated B cells was analyzed by RT-PCR using primers that covered the entire coding sequence of CD137. Three bands were observed, corresponding to the full length CD137 (768 bp) and the two soluble forms of CD137 (sCD137) (636 bp and 502 bp, respectively). These CD137 isoforms are the same as observed in stimulated T cells, meaning that at the mRNA transcription level there is no difference in CD137 expression between activated B and T cells (**Figure 6C**).

### CD137 Signaling Into Primary B Cells Increased TNF and IL-6 Secretion With Increased Cell Proliferation

**Figure 7:**
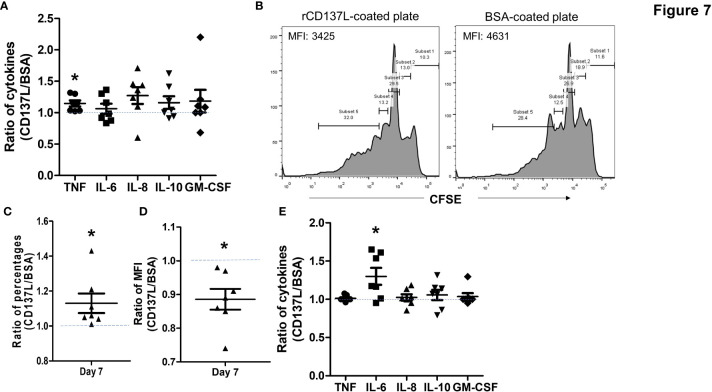
CD137 signaling into B cells induces TNF and IL-6 secretion with increased cell proliferation. CFSE-labeled B cells from seven healthy donors were stimulated with 1 μg/ml of anti-human CD40 antibody + 10 μg/ml of AffiniPure F(ab’)_2_ Fragment Goat Anti-Human IgA + IgG + IgM (H+L) and then cultured in plates coated with 5 μg/ml of rCD137L protein or BSA for up to 7 days. BSA-coated plates served as negative controls. CFSE positivity was based on percentages of cells undergoing at least one cell division. Normalized ratios of cytokine levels, percentages of proliferated cells and CFSE MFI of rCD137L-activated B cells compared to those of BSA-control cells for each donor are shown. Ratios were used to compensate for donor variability. **(A)** Significant increases in TNF secretion from stimulated B cells were observed upon rCD137L activation at 24 h. **(B)** Histograms of CFSE dilution of rCD137L-activated B cells and BSA-treated control cells of one donor are shown. A significant increase in the percentages of proliferated B cells **(C)** and reduction in CFSE MFI of the B cell population **(D)** was observed upon rCD137-activation after 7 days. **(E)** Prolonged secretion of IL-6 was observed upon rCD137L activation up to 7 days. Results are pooled from seven donors (one experiment per donor) and all measurements were done in duplicates. MFI, mean fluorescence index. Each symbol represents one donor. Lines and bars are illustrated means ± standard errors. A value of 1 (dotted line) indicates no difference between rCD137L treatment and control. *p < 0.05 using two-tailed unpaired t test.

In order to determine the functional significance of CD137 expression in primary B cells, B cells from seven healthy donors were stimulated to induce CD137 expression, and then CD137 was engaged by rCD137L for 24 h. A small but significant increase in TNF secretion at 24 h was observed (**Figure 7A**). In addition, CD137 engagement increased proliferation of B cells, as seen by significant increases in the percentages of proliferated cells and drops in CFSE fluorescence of the B cell populations upon rCD137L activation (**Figures 7B–D**). Prolonged CD137 signaling into B cells led to an increased secretion of IL-6 at day 7 (**Figure 7E**). These results illustrate that upon CD137 signaling, B cells release the pro-inflammatory cytokine TNF and IL-6, with an increase in cell proliferation.

### Increased Infiltration of CD137-Expressing B Cells Into Human Brain Organoids

**Figure 8:**
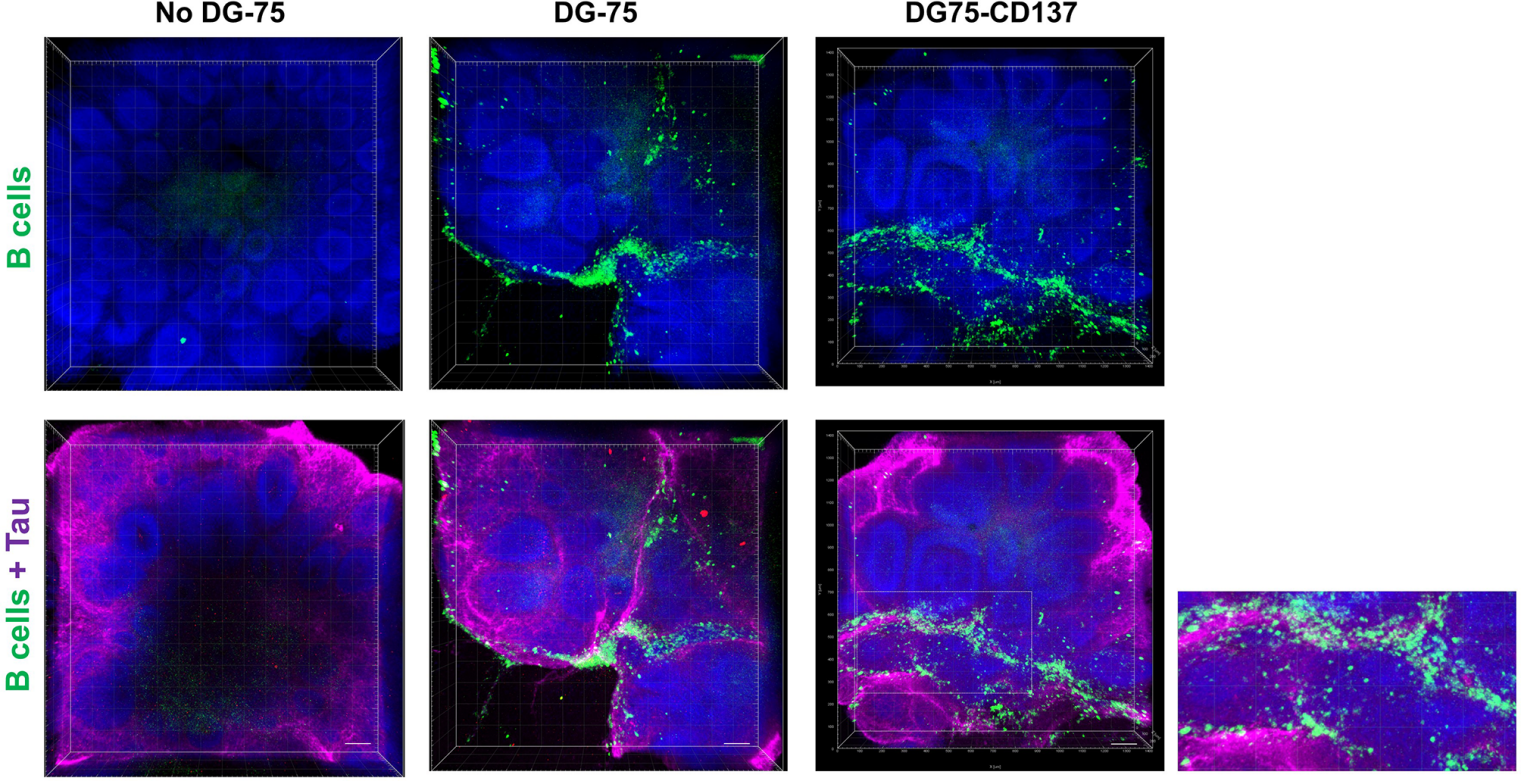
Increased CD137^+^ B cells infiltration into the outermost layers of the rosettes in human brain organoids. Equal amounts of CFSE labeled DG75-CD137 and DG-75 cells (300,000 cells in 2 ml of media) were co-cultured with D42 organoids for 48 h. Two Organoids without DG-75 co-culture served as negative controls. Labeled DG-75 cells (Green) and Tau (Purple) were imaged using confocal microscope. Scale bar = 100 µm. Experiments have been performed twice with consistent results.

Since CD137^+^ B cells were found accumulating in meningeal infiltrates, we studied how CD137^+^ B cells infiltrate the brain using the human brain organoid model. CD137^+^ B cell lines infiltrated and were found surrounding the outermost layers of the rosettes while CD137^-^ parental cells were more scattered with reduced infiltration (**Figure 8**). These results suggest that CD137 expression may enhance the infiltration of B cells into the meninges. **Supplementary Video 1** **and** **2** show 3D videos of images in **Figure 8**, for control B cells and CD137-expressing B cells, respectively.

## Discussion

CD137/CD137L interaction has been shown to contribute to the pathogenesis of EAE (18, 27). This study is the first to demonstrate the presence of CD137^+^ cells in MS brain FFPE samples. CD137^+^ cells were found in both the brain parenchyma and brain blood vessels, and most CD137^+^ cells were identified at sites of active inflammation with demyelination. The significantly higher number of CD137^+^ cells in active MS lesions suggests a contribution of CD137^+^ cells to the inflammatory process, since CD137 is a potent costimulatory molecule expressed upon cell activation. There are two previous studies reporting higher levels of soluble CD137 (sCD137) in MS patients’ sera compared to those of healthy controls. In patients with active MS, there is intrathecal release of sCD137 leading to high levels of sCD137 in cerebrospinal fluid and sera (11, 28). sCD137 is produced by alternative splicing at the level of transcription, and its level parallels with that of membrane-bound CD137 (10). CD137 expression identified in this study is found both intracellularly and extracellularly, and sCD137 produced intrathecally during active MS may come from these CD137^+^ cells.

This study is the first to identify CD137 expression on B cells in autoimmune disease. These CD137^+^ B cells are present in leptomeningeal infiltrates and in diffuse white matter lesions. Both BCR and CD40 signaling are required for CD137 expression to be induced on B cells. However, we observed a significant drop of CD137 expression on B cells after 48 h of stimulation if no additional stimuli were added, suggesting that CD137 expression on B cells is also transient.

In MS, autoreactive B cells are activated by neural antigens. Activated antigen-specific T cells express CD40L which can interact with CD40 on activated B cells (29). Our *in vitro* experiment on B cell lines and primary B cells mimic these two signals in inflammation. An increase in TNF secretion from stimulated B cells upon rCD137L activation was observed at 24 h. *In vitro* experiments of B cell lines showed high levels of IL-10 secretion upon transduction with CD137, and a further increase upon enhancing CD137 signaling by rCD137L, which was likely a reflection of cell activation, supported by the observation of increased CD40 expression upon CD137 signaling (30). This phenomenon was not observed in primary B cells upon CD137 signaling. Instead, IL-6 secretion was significantly enhanced upon activation by rCD137L while IL-10 secretion remained similar between treatment and control. IL-6 is known to promote Th1 and Th17 polarisations in EAE. IL-6-deficient mice are protected against EAE development, and IL-6 produced by B cells plays a role in EAE progression (31, 32). A cytokine imbalance of increased IL-6 secretion and decreased IL-10 secretion by PBMCs is found in MS, though the exact cause remains unclear (33). CD137 signaling into B cells may help to shift the balance of cytokines toward a pro-inflammatory status, thus enhancing inflammation.

Besides enhanced cytokine secretion, B cell proliferation increases upon CD137 signaling. Our results are consistent with a previous study by Nakaima et al., which focused on the proliferative capacity of B cells in patients with CLL. Higher CD137 expression on B cells of CLL patients is observed when compared to other malignancies and healthy controls, and increased B cell proliferation upon CD137 signaling is mediated by the NF-κB pathway (24). In MS, increased B cell proliferation may facilitate the aggregation of meningeal infiltrates. In this study, multiplex staining of MS lesions demonstrates the presence of CD137^+^ B cells in meningeal infiltrates. It suggests that CD137 expression may be induced in compartmentalized inflammation in MS. In meningeal infiltrates, clusters of B cells can be found which resemble B follicles in germinal centers of secondary lymphoid organs (34). Magliozzi et al. have reported increased levels of inflammatory cytokines in the CSF of MS patients with high level of meningeal inflammation, and many of these cytokines are related to B cell activity including TNF, IL-6, IL-10, CXCL13, CXCL10, and LT-α (35). As shown by functional experiments, CD137 signaling into B cells leads to clonal expansion and prolonged IL-6 secretion, which may further promote proliferation and accumulation of B cells in meningeal infiltrates. This hypothesis is further supported by CD137^+^ B cells infiltrating at a higher rate into human brain organoids than CD137^-^ B cell. The exact reason of increased infiltration still needs further investigation.

In this study, CD137 expression is identified on blood vessel walls in MS, but not in those of healthy controls. This is consistent with a previous report by Drenkard et al., which demonstrates CD137 expression on inflamed blood vessel walls of patients with various autoimmune conditions (9). IL-1 and TNF secretion induces CD137 expression on endothelial cells and CD137 expressed on blood vessel walls enhances rolling and adherence of circulating monocytes into the site of inflammation (23). In MS, CD137 expression on the endothelial lining may enhance the recruitment of circulating CD137L^+^ monocytes to the site of inflammation.

For some of the CD137^+^ cells, the cell types remain yet to be identified. These cells may be leukocytes, judging from the cell sizes and morphologies. CD137 expression on CD3^+^ T cells was not frequently found. This is in line with another study characterizing T cell phenotypes and functions, where CD137^+^ CD8^+^ T cells are only found in lesions with diffuse white matter abnormalities, but not in other types of MS lesions including active, mixed active/inactive and inactive lesions (36).

This study demonstrates the presence of CD137 expression in MS brain tissues, and that the number of CD137^+^ cells is highest in active lesions. It identifies, for the first time, the presence of CD137^+^ B cells in an autoimmune disease, and provides evidence for a functional role of CD137 signaling into B cells in the inflammatory process.

## Data Availability Statement

Requests to access the datasets should be directed to phssh@nus.edu.sg.

## Author Contributions

HW planned the study, performed the experiments, analyzed the data, and wrote the manuscript. AP and JJEC planned and performed the experiments of organoids. SG generated the CD137-expressing lentivirus. HS planned the study, wrote the manuscript, and supervised the study. All authors contributed to the article and approved the submitted version.

## Funding

This study was funded by the Ministry of Education, Singapore (MOE2019‐T2‐2‐087). Funding support from iHealthtech to JJEC is acknowledged.

## Conflict of Interest

The authors declare that the research was conducted in the absence of any commercial or financial relationships that could be construed as a potential conflict of interest.
