# Supplementary material for: Identification of CD137-Expressing B Cells in Multiple Sclerosis Which Secrete IL-6 Upon Engagement by CD137 Ligand
Source: Front Immunol. 2020 Nov 6;11:571964. doi: 10.3389/fimmu.2020.571964 (PMC7677239; doi:10.3389/fimmu.2020.571964)
Supplement: Supplementary file 1 [file DataSheet_1.pdf]

Supplementary Table 1

Patient	Age (yr)	Sex	Duration of disease (yr)	Type of MS	Treatment Received
MS058	51	F	21	SPMS	Steroids
MS094	42	F	6	PPMS	N/A
MS103	77	F	21	SPMS	N/A
MS121	49	F	14	SPMS	N/A
MS122	45	M	10	PPMS	Steroids
MS124	30	F	5	SPMS	N/A
MS136	40	M	9	SPMS	Beta Interferon
MS153	50	F	N/A	SPMS	N/A
MS154	34	F	11	SPMS	N/A
MS160	44	F	16	SPMS	N/A
MS166	52	F	26	SPMS	N/A
MS168	88	F	30	PPMS	N/A
MS169	80	F	42	SPMS	N/A
MS170	56	M	26	PPMS	N/A
MS179	70	F	N/A	SPMS	N/A
MS180	44	F	N/A	SPMS	Steroids
MS182	56	F	13	PPMS	Steroids
MS186	58	F	37	SPMS	N/A
MS187	57	F	18	SPMS	Steroids
MS195	47	F	27	SPMS	Steroids
MS212	47	F	29	SPMS	N/A
MS216	53	F	N/A	PPMS	N/A
MS273	61	M	31	PPMS	N/A
MS325	51	M	2	PPMS	N/A
MS330	59	F	39	SPMS	N/A
MS336	57	F	26	SPMS	N/A
MS340	53	F	19	SPMS	N/A
MS352	43	M	18	SPMS	N/A
MS363	42	M	27	PPMS	N/A
MS383	42	M	8	PPMS	N/A
MS386	90	F	59	PPMS	N/A
MS473	39	F	13	PPMS	N/A
MS485	57	F	29	PPMS	N/A
MS492	66	F	31	PPMS	N/A
MS497	60	F	29	SPMS	Steroids & Beta Interferon
MS513	51	M	19	SPMS	Steroids
CS022	69	F	/	HC	/
CS036	68	M	/	HC	/
CS037	84	M	/	HC	/
CS039	89	M	/	HC	/
CS044	67	F	/	HC	/
CS052	70	M	/	HC	/
CS053	66	M	/	HC	/
CS064	63	F	/	HC	/

Supplementary Table 1. Characteristics of MS patients.

PPMS: Primary progressive Multiple Sclerosis; SPMS: Secondary progressive Multiple Sclerosis; HC: Healthy controls; N/A: Not Available

Supplementary Figure 1

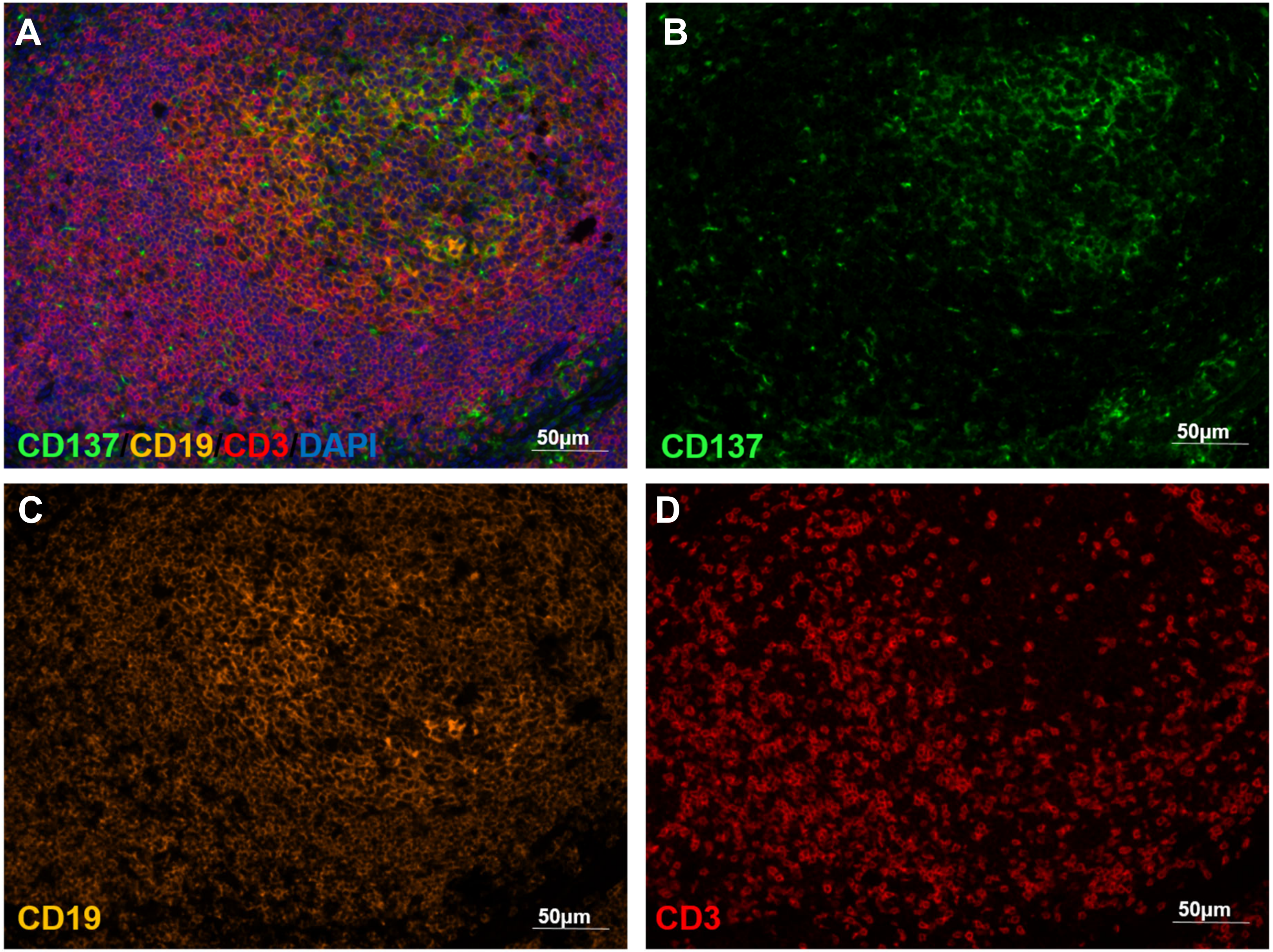

Supplementary Figure 1. CD137, CD19 and CD3 staining in human tonsil tissue. (A) 4-colour multiplex staining showing a germinal center in tonsil. (B-D) represent CD137, CD19 and CD3 staining respectively.

Supplementary Figure 2

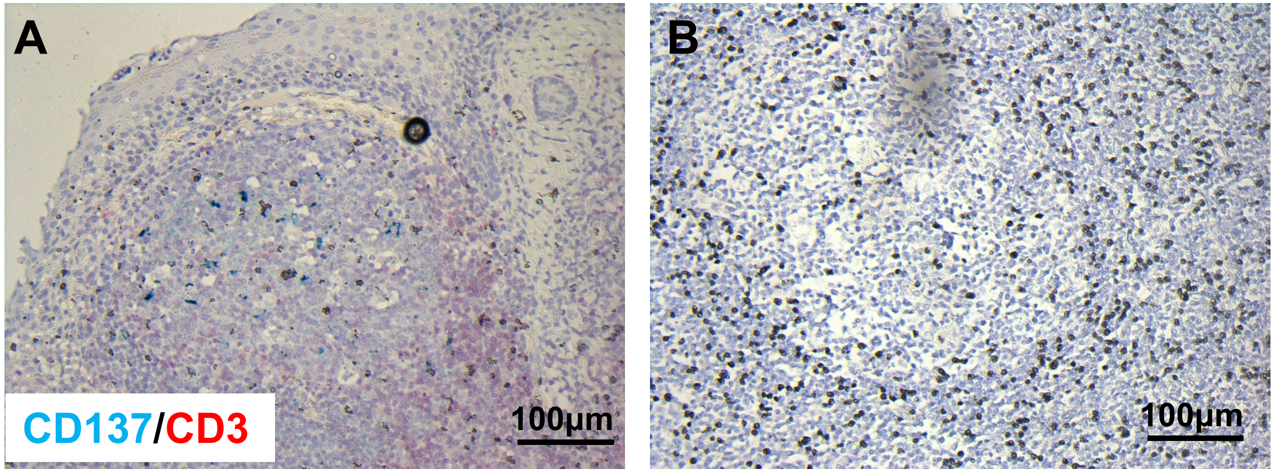

Supplementary Figure 2. CD137 and CD3 double immunohistochemical staining in human tonsil tissue with respective isotype controls. Tonsil slides were first incubated with either (A) mouse anti-human CD137 antibody + rabbit anti-human CD3 antibody or (B) mouse isotype control + rabbit isotype control overnight, followed by incubation with HRP-labelled anti-mouse and anti-rabbit secondary antibodies.

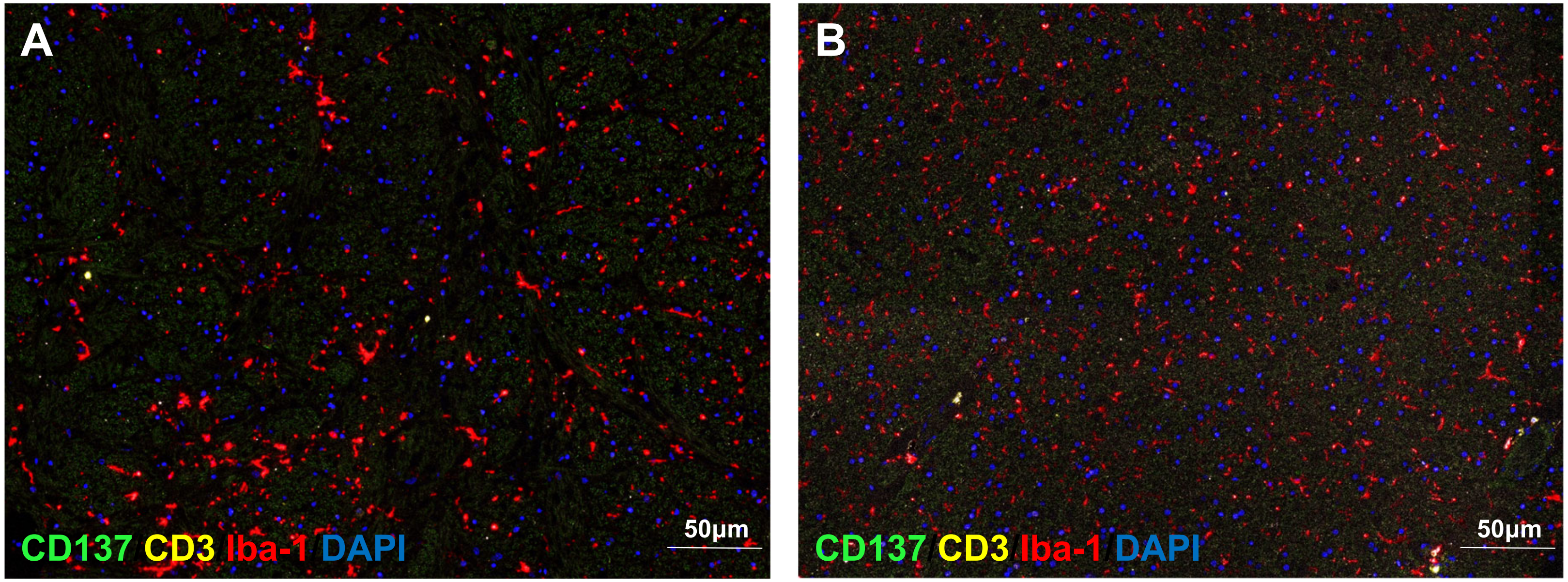

Supplementary Figure 3

Supplementary Figure 3. Representative images from two healthy controls A) CS022 and B) CS053 are shown.

Supplementary Figure 4

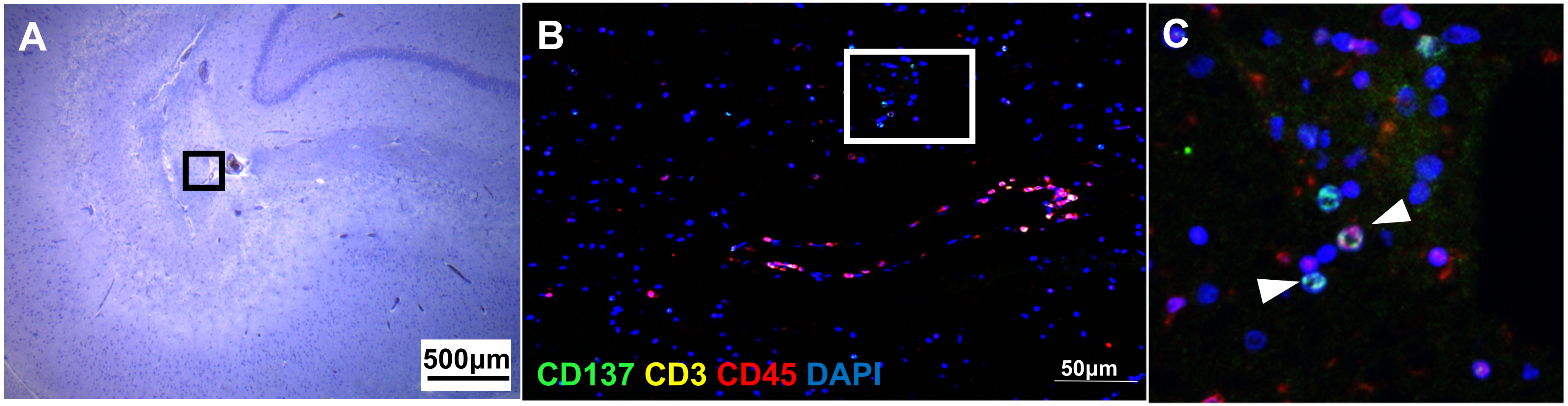

Supplementary Figure 4. CD137⁺ leukocytes in active MS lesion. (A) Luxol and Cresyl staining of a section from the temporal cortex with active lesion (MS 325). (B-C) Opal staining of boxed region in A. CD137⁺ CD45⁺ cells were found at the perivascular region (white arrowheads).

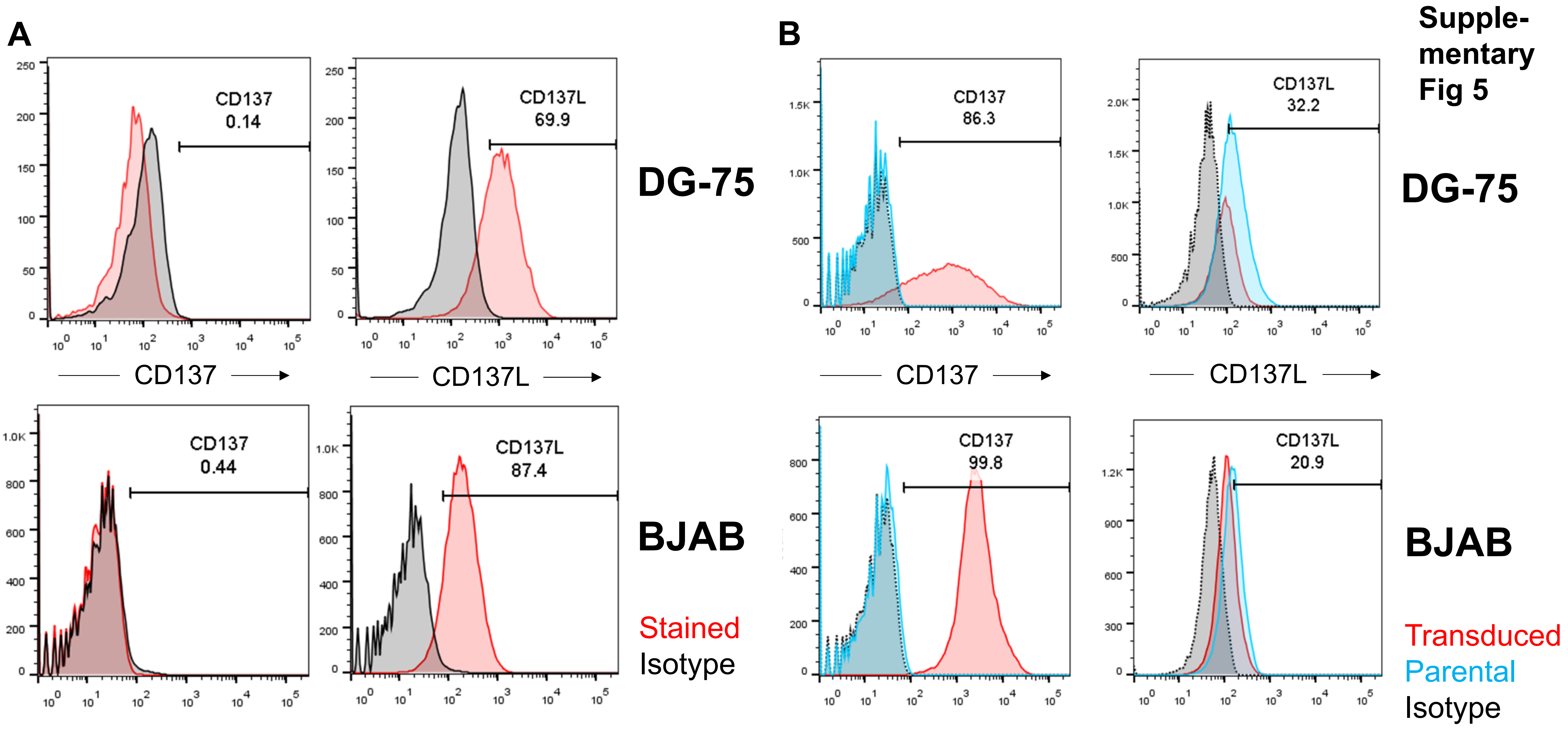

Supplementary Figure 5. CD137 and CD137L expression in transduced and parental B cell lines. Full length cDNA of CD137 was cloned into pLenti6 vector and lentiviral particles were produced. DG-75 and BJAB cells were then transduced and selected with Blasticidin.

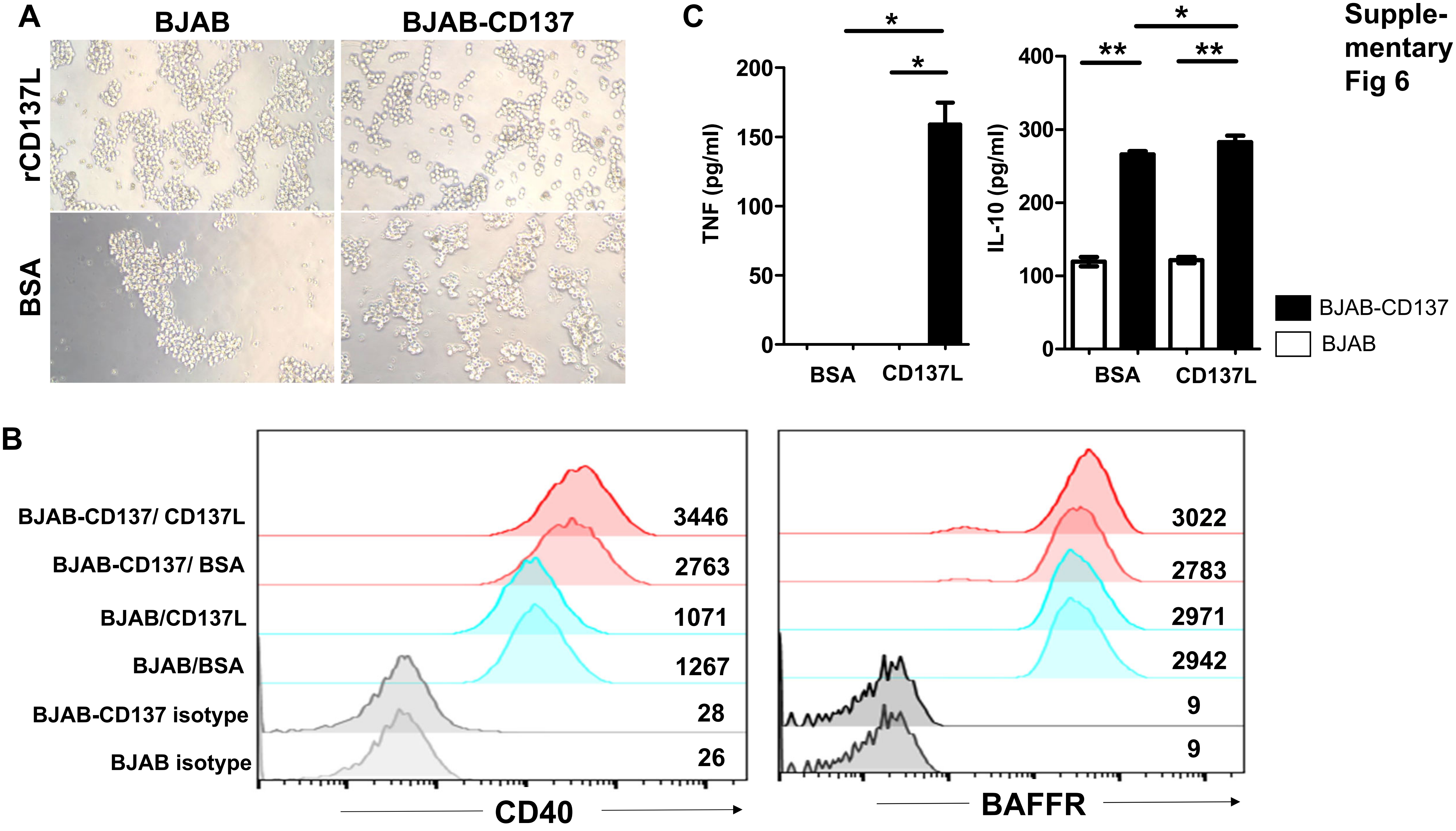

Supple-
mentary
Fig 6

Supplementary Figure 6. CD137 signalling into the B cell line BJAB. (A) Morphologies of BJAB-CD137 and parental BJAB cells cultured in plates coated with 5 µg/ml of either rCD137L or BSA for 24 h. Similar morphologies of cells were observed for BJAB as for DG75 cells upon activation. (B) Expression of CD40 and BAFFR upon activation of BJAB and BJAB-CD137 cells. Numbers in the charts represent mean fluorescence index of the respective populations. All experiments were repeated for three times with consistent results. (C) Levels of TNF and IL-10 in supernatants after 24 h of activation. IL-6 and IL-8 levels were negligible. Results are representative of 3 independent experiments. *$p < 0.05$ and **$p < 0.001$ using two-tailed unpaired t test.
